# Ultrasound-guided transbronchial biopsy in the diagnosis of fibrosing mediastinitis-associated pulmonary hypertension

**DOI:** 10.1186/s13023-025-03695-3

**Published:** 2025-04-15

**Authors:** Yu Zhang, Han-Xiang Song, Yong-Jia Qi, Nan-Nan Sun, Zan-Sheng Huang, Wan-Lei Fu, Jing Zhang, Felix J. F. Herth, Ye Fan

**Affiliations:** 1 Department of Respiratory Disease, Xinqiao Hospital, Third Military Medical University, Chongqing, China; 2 Department of Pathology, Xinqiao Hospital, Third Military Medical University, Chongqing, China; 3 Chongqing Public Health Medical Center, Chongqing, China; 4 Department of Pneumology and Critical Care Medicine, Thoraxklinik, and Translational Lung Research Center Heidelberg, University of Heidelberg, Heidelberg, Germany

**Keywords:** Fibrosing mediastinitis, Pulmonary hypertension, Endobronchial ultrasound, Cryobiopsy, Needle aspiration

## Abstract

**Background:**

Fibrosing mediastinitis is a rare benign disease frequently complicated by pulmonary hypertension. A definitive diagnosis for fibrosing mediastinitis-associated pulmonary hypertension (FM-PH) and its etiologies necessitates mediastinal biopsy and subsequent pathological assessment. Endobronchial ultrasound (EBUS)-guided transbronchial mediastinal cryobiopsy is a recently developed technique that provides diagnostic advantages over standard needle biopsy, particularly in benign mediastinal disorders. Nevertheless, their safety and efficacy in diagnosing FM-PH remain elusive.

**Methods:**

We retrospectively studied patients with mediastinal lesion and pulmonary vascular compression who underwent both transbronchial needle aspiration and mediastinal cryobiopsy with EBUS guidance. Diagnostic yields of FM-PH and its etiologies, along with procedure-related adverse events, were analyzed. Immunohistochemical study was conducted to identify immunological properties of FM-PH.

**Results:**

Of the 529 patients with mediastinal lesions, 80 exhibited pulmonary vessel compression, including 10 who were ultimately diagnosed with FM-PH following mediastinal biopsy and right heart catheterization. Cryobiopsy showed a higher diagnostic yield for FM-PH compared to needle aspiration (100% versus 40%, *p* = 0.011). Disease etiologies included pneumoconiosis in 5 cases, tuberculosis in 3, and idiopathic FM-PH in the remaining 2. Cryobiopsy appeared to be superior to needle biopsy for etiological diagnosis, although this difference was not statistically significant (80% versus 60%, *p* = 0.628). Immunohistochemical analyses of cryosamples revealed mixed inflammatory infiltrates of B and T lymphocytes, as well as macrophages, surrounding or within FM-PH lesions. There was no significant bleeding or other complications.

**Conclusion:**

Transbronchial mediastinal cryobiopsy might be a safe and effective diagnostic tool for FM-PH, offering valuable information for personalized treatment.

**Supplementary Information:**

The online version contains supplementary material available at 10.1186/s13023-025-03695-3.

## Background

Pulmonary hypertension is a rare but fatal disease characterized by abnormally elevated pressure and resistance of the pulmonary circulation, resulting in right heart failure and patient death if not well managed [1–3]. Fibrosing mediastinitis (FM), also regarded as an orphan disease, could lead to pulmonary hypertension via compression or occlusion of mediastinal vascular structures caused by locally invasive fibrous tissue within the mediastinum [4–6]. The etiology of fibrosing mediastinitis can be either idiopathic or linked to multiple factors, such as radiation, infections, and systemic diseases like sarcoidosis. The most common aetiological factors for FM are infection with Histoplasma capsulatum in USA, while Mycobacterium tuberculosis is one of the major causes in East Asia [6–8]. Currently, the prognosis of fibrosing mediastinitis-associated pulmonary hypertension (FM-PH) is not optimistic, primarily due to the lack of accurate diagnosis and effective treatment.

Definitive diagnosis of FM is traditionally achieved through surgical exploration and pathological biopsy, using either mediastinoscopy or thoracotomy. Nonetheless, these procedures are often restricted by their invasive nature and the significant risk of complications [9, 10]. Despite being the leading approach for mediastinal sampling, endobronchial ultrasound-guided transbronchial needle aspiration (EBUS-TBNA) yields limited sample sizes, which might hamper its ability to diagnose benign lesions that are the main causes of FM-PH, including tuberculosis, sarcoidosis, and pneumoconiosis [11–13]. Transbronchial mediastinal cryobiopsy is a newly developed minimally invasive procedure offering larger and better-preserved mediastinal specimens compared to traditional needle biopsies, improving the accuracy of diagnosing various conditions, particularly benign disorders and rare tumors [14, 15]. In this study, we assessed the safety and efficacy of ultrasound-guided transbronchial biopsy for obtaining pathological and etiological diagnoses of FM-PH. Additionally, we further characterized the immunological properties of this disease.

## Methods

### Study design

We conducted a retrospective review of patients with mediastinal lesions who were admitted to Chongqing Xinqiao Hospital for diagnostic bronchoscopy between 2019 and 2024. Inclusion criteria were as follows: (1) age greater than 18 years, (2) presence of at least one mediastinal lesion suspected of causing compression of mediastinal vascular structures, as identified on contrast-enhanced CT or PET-CT, (3) pathological evaluation of biopsy samples obtained through EBUS-guided procedures confirming a diagnosis of FM, (4) right heart catheterization showing a mean pulmonary artery pressure greater than 20 mmHg. The exclusion criteria were: (1) needs for additional procedures other than EBUS (such as endobronchial biopsy), (2) a final diagnosis of non-FM mediastinal lesion, and (3) no evidence of pulmonary hypertension. All clinical data were pseudonymized to ensure the confidentiality of patients. Written informed consent was obtained from all participants, and this study was approved by the Ethics Committee of Third Military Medical University (2019 - 062- 01).

### EBUS procedure

All participants underwent EBUS investigation using an ultrasound bronchoscope (BF-UC260 F-OL8 or BF-UC260 F, Olympus, Tokyo, Japan), operated by an experienced bronchoscopist. The airway was first examined using endoscopy and ultrasonography. Upon identification of mediastinal lesions, their location, size, and vascular supply were recorded. Four needle aspirations were then performed, followed by one cryobiopsy, as priorly described [16]. Pathological specimens were subsequently evaluated by a pathologist. For immunological study, remaining tissue samples obtained for pathological diagnosis were retrieved, and consecutive sections were stained using antibodies against CD3, CD4, CD8, CD20, CD56, CD68, BCL6, FOXP3, and PD- 1 (**Supplement**).

### Right heart catheterization

Right heart catheterization was performed in patients diagnosed with FM through EBUS-guided biopsies, who exhibited pulmonary vascular compression or obstruction due to mediastinal lesions on CT pulmonary angiography, and/or radiographic or echocardiographic signs suggestive of pulmonary hypertension (e.g., enlarged pulmonary artery diameter [PA-to-aorta ratio > 0.9], increased systolic peak tricuspid regurgitation velocity > 2.8 m/s, et al.). Patients were positioned supine, and local anesthesia was administered. A successful femoral venous puncture was done, followed by the insertion of an 8 F vascular sheath. A Swan-Ganz catheter was advanced through the sheath into the inferior vena cava, right atrium, right ventricle, and pulmonary artery for hemodynamic assessment, including right atrial pressure, right ventricular pressure, pulmonary artery pressure, pulmonary capillary wedge pressure, and cardiac output. Post-procedure, the catheter and sheath were removed, and hemostasis was achieved by compression bandaging. Vital signs and access site integrity were monitored for 6 h.

### Histopathologic definition of FM

The histopathologic diagnosis of FM was based on the findings from a prior study, specifically the presence of dense, paucicellular fibrous tissue infiltrating and obliterating adipose tissue, with or without patchy mononuclear cell infiltrates [7]. Patients with benign or malignant mediastinal tumors were excluded.

### Adverse events

All patients received post-procedural chest radiography and were monitored for adverse events related to the EBUS procedure, such as airway bleeding, pneumothorax, pneumomediastinum, and mediastinitis, for 4 weeks following mediastinal biopsy. Bleeding was classified based on criteria defined in prior studies: grade 0, traces of blood not requiring suctioning; grade 1, bleeding only requiring suctioning and hemostatic wedging for up to 2 min (two 1-min cycles); grade 2, bleeding requiring hemostatic wedging for 3 min or more; grade 3, bleeding requiring topical instillation of epinephrine or ice cold saline; and grade 4, bleeding requiring hemodynamic support, transfusion of blood products, selective mainstem intubation, bronchial blocker, hospital admission, or surgical intervention [17, 18].

### Study outcomes

The primary endpoint was the overall diagnostic yields for FM-PH and its associated disease etiology, as well as the yield for each biopsy method. A biopsy was considered diagnostic if the pathological assessment yielded a definitive diagnosis. Any findings that were vague or not clearly defined were classified as nondiagnostic. Follow-up results were not factored into the calculation of the diagnostic yield. The secondary endpoints included adverse events and immunological findings.

### Statistical analysis

Data were expressed as mean ± SD and analyzed using PASW Statistics 27 (SPSS Inc., Chicago, IL, USA). Statistical comparisons were conducted using Chi-squared test. A *p*-value of < 0.05 was considered statistically significant.

## Results

### Patients

**Fig. 1:**
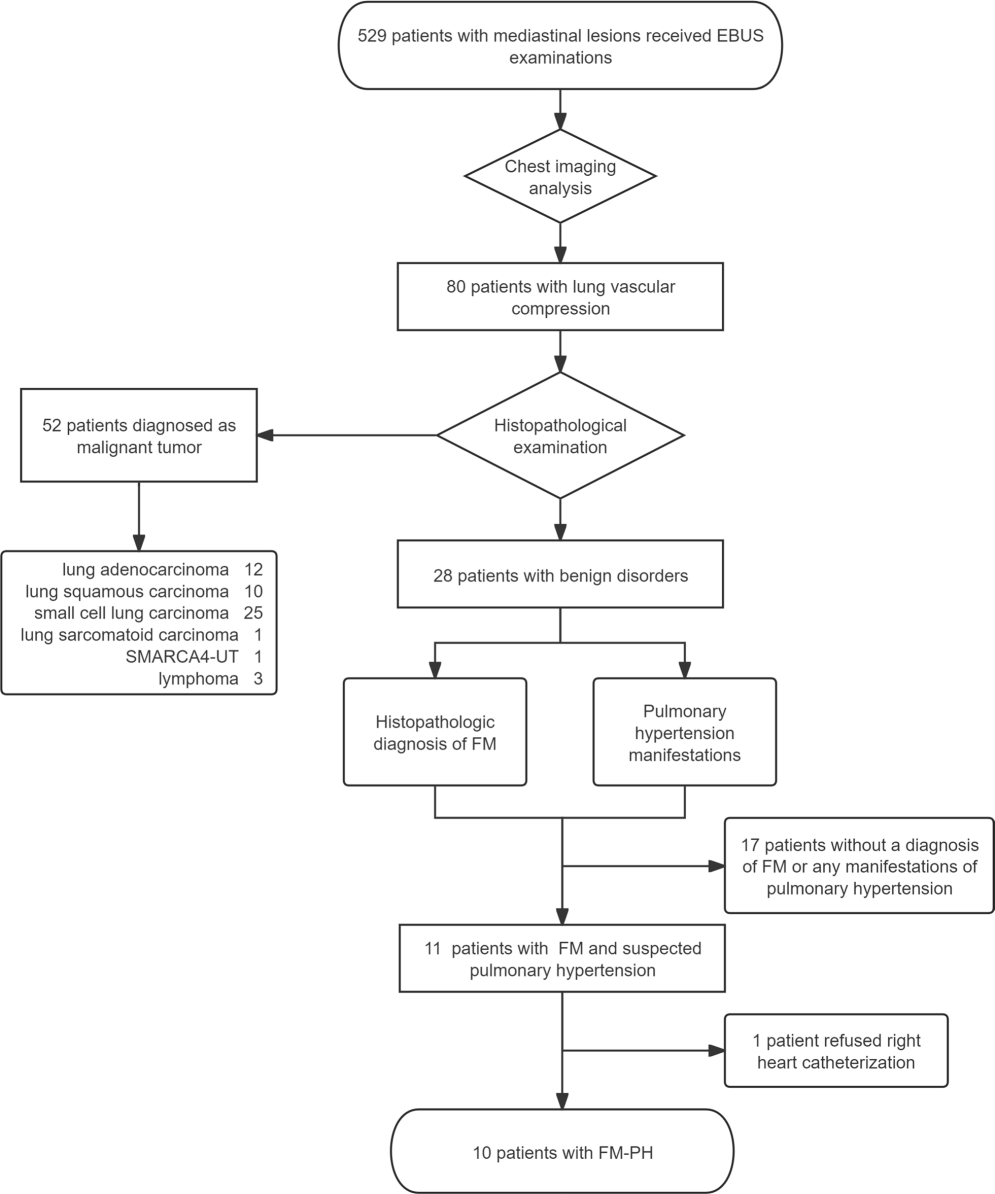
Patient flow. EBUS, endobronchial ultrasound

Between Oct 2019 and June 2024, 529 patients with mediastinal lesions underwent diagnostic bronchoscopy at Chongqing Xinqiao Hospital (Fig. 1). Imaging analysis using contrast-enhanced CT or PET-CT revealed pulmonary vascular compression in 80 of these patients. Among them, 10 were finally diagnosed with FM-PH following pathological examination and right heart catheterization. Fifty-two patients were excluded due to a malignant diagnosis, while 17 were excluded either because of a non-FM diagnosis or the absence of pulmonary hypertension manifestations. Additionally, 1 patient was not included because of a refusal to receive right heart catheterization. All participants tolerated EBUS procedures with moderate sedation well, and each biopsy was technically successful. Table 1 details the baseline characteristics of the patient cohorts. Participants had a median age of 63.4 years, and 4 (40%) of them were women. The most common symptoms reported were cough (n = 10, 100%), dyspnea (n = 6, 60%), chest pain (n = 3, 30%), peripheral edema (n = 2, 20%), and hemoptysis (n = 1, 10%). Each patient presented with a combination of symptoms.

**Table 1:** Patient baseline characteristics

	FM-PH
Age (year)	63.4 (9.0)
*Sex*	
Female	4 (40.0)
Male	6 (60.0)
BMI (kg/m^2^)	24.3 (5.6)
Smoking (pack-year)	5 (8.5)
*Lesion extent*	
Focal	5 (50.0)
Diffuse	5 (50.0)
*Pulmonary vessel compression*	
Artery	10 (100.0)
Vein	3 (30.0)
Both	3 (30.0)
Systolic pulmonary artery pressure (mmHg)	66.2 (24.0)
Mean pulmonary artery pressure (mmHg)	39.2 (13.3)

Data are mean (SD) or n (%). FM-PH, fibrosing mediastinitis-associated pulmonary hypertension. BMI, body mass index

### Chest imaging

**Fig. 2:**
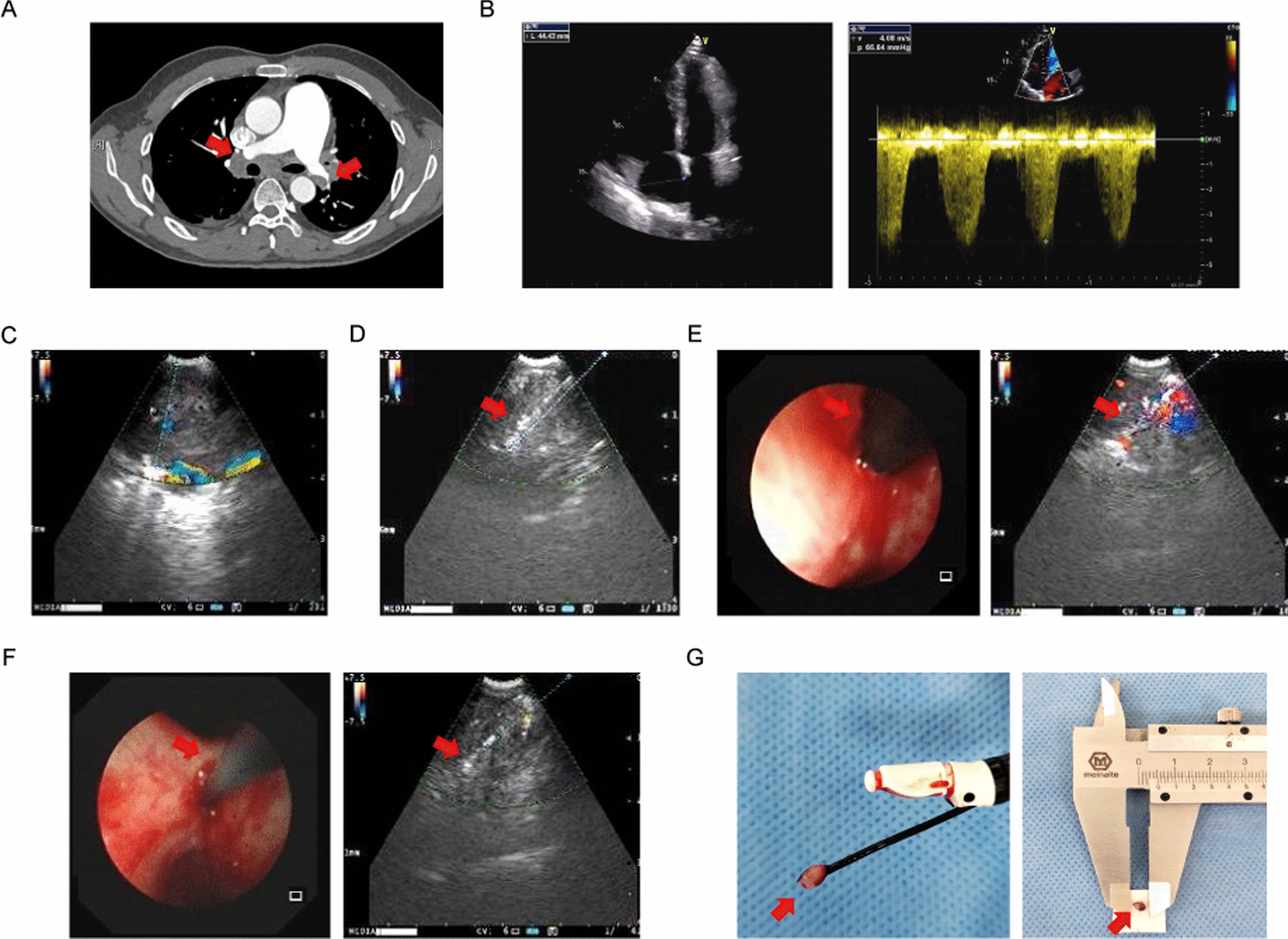
**A** CT pulmonary angiogram shows mediastinal lesions and compression of the pulmonary vessels (red arrow). **B** Echocardiograph reveals enlarged right ventricle, compression of left heart, and elevated right ventricular systolic pressure, indicating pulmonary hypertension. **C** Endobronchial doppler image shows the blood flow of the mediastinal mass. **D** EBUS-TBNA (red arrow) biopsy of mediastinal lesion. **E** Transbronchial incision was made with a high-frequency needle knife (red arrow) to create a working channel for the cryoprobe under EBUS monitoring **F** EBUS-guided transbronchial mediastinal cryobiopsy was performed by advancing the cryoprobe (red arrow) into the lesion. **G** Mediastinal specimen (red arrow) was obtained by transbronchial mediastinal cryobiopsy

Chest CT scans detected focal mediastinal lesions in 5 of the 10 patients (50%), while the remaining 5 (50%) showed diffuse mediastinal infiltration (Fig. 2). Mediastinal abnormalities localized to the right side causing right pulmonary vessel constriction in 4 patients (40%), and bilateral involvement was found in the other 6 patients (60%). All patients (100%) experienced constriction of the pulmonary arteries, while 3 (30%) had compression of both the pulmonary arteries and veins. Calcifications were noted in 7 patients (70%).

### Pathological diagnosis

The biopsy results are summarized in Table 2. All acquired materials were deemed suitable for histopathologic examination. A diagnosis of FM could be achieved through both TBNA and cryobiopsy in 4 patients (40%). However, needle aspiration failed to offer a conclusive diagnosis of FM in the remaining 6 cases (60%), which could only be established from cryobiopsy (40% versus 100%, *P* = 0.011).

**Table 2:** Diagnostic yield analyses

	TBNA	Cryobiopsy	*P* value
Diagnostic yield (FM-PH)			0.011
No	6 (60.0)	0 (0.0)	
Yes	4 (40.0)	10 (100.0)	
Diagnostic yield (Etiology)			0.628
No	4 (40.0)	2 (20.0)	
Yes	6 (60.0)	8 (80.0)	
Diagnostic yield (Tuberculosis)			1
No	1 (20.0)	0 (0.0)	
Yes	4 (80.0)	5 (100.0)	
Diagnostic yield (Pneumoconiosis)			1
No	1 (33.3)	0 (0.0)	
Yes	2 (66.7)	3 (100.0)	

Data are n (%). TBNA, transbronchial needle aspiration. FM-PH, fibrosing mediastinitis-associated pulmonary hypertension

### Etiological diagnosis

In 8 patients (80%), the mediastinal specimens led to a definite etiological diagnosis. Of those, 5 were diagnosed as pneumoconiosis and 3 as tuberculosis. The remaining 2 cases were classified as idiopathic FM, with histopathological analyses revealing hyperplastic fibrous tissue without granuloma. Consistent with prior studies, transbronchial mediastinal cryobiopsy demonstrated a higher diagnostic yield for benign disorders relative to traditional needle aspiration, although this trend did not reach statistical significance (80% versus 60%, *P* = 0.628) [14, 15].

### Adverse event

There were no severe complications during EBUS procedures or at the 4-week follow-up (Table 3). The most frequently observed adverse event was mild to moderate bleeding, which required no interventions beyond local hemostatic measures, such as topical instillation of epinephrine or ice cold saline. All patients received post-procedure chest radiograph, and no pneumothorax or pneumomediastinum was detected. One patient died of chronic myeloid leukemia 6 months after discharge.

**Table 3:** Procedural complications

	EBUS procedure
*Bleeding*	
Grade 1	5 (50.0)
Grade 2	2 (20.0)
Grade 3	2 (20.0)
Grade 4	0 (0.0)
Pneumothorax	0 (0.0)
Pneumomediastinum	0 (0.0)
Death	0 (0.0)

Data are n (%). FM-PH, fibrosing mediastinitis-associated pulmonary hypertension. BMI, body mass index

### Immunological characteristics

**Fig. 3:**
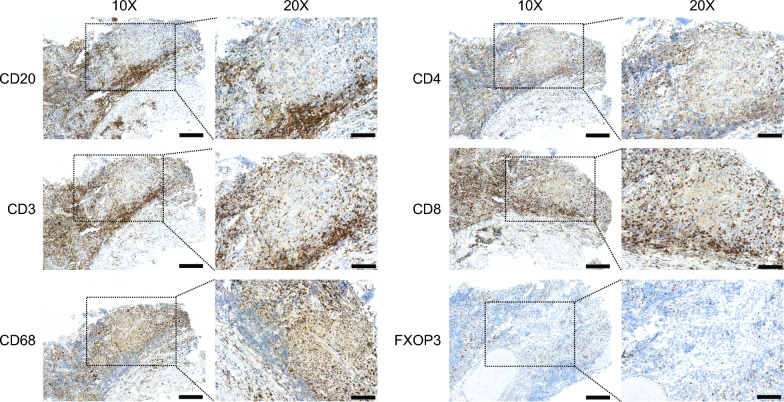
Immunohistochemical staining of cryosample (CD20: B cell marker, CD3: T cell marker, CD4: T helper cell marker, CD8: cytotoxic t cell marker, CD68: macrophage marker, FOXP3, regulatory t cell marker). Scale bars = 60 or 100 μm

The adaptive immune responses within mediastinal lesions of FM-PH patients were evaluated through immunostaining study, which suggested the presence of mixed inflammatory infiltrates (Fig. 3). In line with prior studies, we observed a prominent infiltration of CD20^+^ B lymphocytes predominantly surrounding the fibrotic lesions [7]. CD3^+^ T lymphocytes were found both adjacent to and sporadically distributed within the fibrotic lesions, with a slightly higher proportion of CD4^+^ cells relative to CD8^+^ cells. Unlike lymphocytes, macrophages were mainly present within the fibrotic area, rather than at the periphery of the lesions. Interestingly, we noted the accumulation of FOXP3^+^ cells around the fibrotic lesion and small vessels. The staining for CD56, BCL6, and PD- 1 was minimally detected in both the fibrotic areas and peripheral regions.

## Discussion

FM features extensive proliferation of locally invasive fibrous tissue, which encases organs and compresses bronchovascular structures within the mediastinum, frequently leading to respiratory and vascular abnormalities. Pulmonary hypertension caused by progressive narrowing of lung vessels is the primary cause for mortality linked to FM [13, 19]. Nevertheless, a major challenge in improving the prognosis of FM-PH lies in the complexity of obtaining a timely and accurate histopathologic diagnosis. Moreover, the multifactorial nature of FM-PH—encompassing factors such as irradiation, infection, and systemic inflammatory disorders—highlights the essentiality of understanding disease etiology, which is necessary for developing effective, personalized therapeutic strategy [20].

While contrast-enhanced CT and right heart catheterization are normally required for the clinical diagnosis of FM-PH, mediastinal biopsy is crucial for a definitive pathological diagnosis and the exclusion of secondary causes. Current recommendations are to favor surgical procedures over needle biopsy, as extensive sampling is necessary for confidently ruling out fibrosis-producing neoplasms, such as sclerosing non-Hodgkin lymphoma or nodular sclerosis variant of Hodgkin disease [8, 21]. Accordingly, the largest meta-analysis on diagnostic efficacy of EBUS-TBNA for lymphoma reveals a pooled sensitivity of only 66.2% [22]. The limitations of needle-based techniques in FM-PH might also extend to etiological diagnosis, as they often yield limited samples that are suitable solely for cytologic evaluation, making the diagnosis of non-lung cancer mediastinal lesions particularly challenging [23–25]. Nevertheless, it is worth noting that surgical sampling performed during mediastinoscopy, thoracoscopy, or open thoracotomy could pose significant risks for FM-PH patients with compromised health or impaired cardiorespiratory function [10].

Driven by the ability of cryobiopsy to yield large, well-preserved lung specimens, we recently employed cryoprobe as an innovative sampling technique for diagnosing mediastinal disease. This technique, providing the greatest volume of intact mediastinal tissue compared to other minimally invasive biopsy approaches, enables more comprehensive histopathological analysis and supports advanced molecular and immunological evaluations [14–16]. Randomized controlled trials have demonstrated that transbronchial mediastinal cryobiopsy offers diagnostic advantages over standard needle aspiration, especially for benign diseases, many of which are underlying etiologies of FM-PH [15, 26]. In line with these findings, our study demonstrated the potential benefits of mediastinal cryobiopsy in FM-PH, serving not only for pathological confirmation but also for etiological investigation.

Importantly, the diagnostic advantage of mediastinal cryobiopsy was achieved without compromising patient safety, as no serious perioperative complications were reported in this cohort. We opt for conscious sedation due to its proven tolerability and safety in more than 700 mediastinal cryobiopsy cases, avoiding the potential side effects of general anesthesia [14, 15]. There are concerns regarding bleeding risks of mediastinal cryobiopsy in FM-PH patients, mainly because of its high intravascular pressure and extensive collateral circulation [6, 7]. Intriguingly, we observed no significant hemorrhagic events during cryobiopsy, which might be attributed to the coagulative effects of electrocautery during airway incision and the strategic avoidance of vascular-rich areas, guided by Doppler ultrasound.

Both surgical treatments and non-surgical procedures, such as endovascular interventions, effectively relieve the compression of mediastinal structures caused by proliferative fibrous tissues and help ameliorate the related symptoms [4, 6, 27, 28]. However, surgical therapies are reported to be associated with high peri- and postoperative mortality exceeding 30%, and most patients treated with endovascular procedures experience stent restenosis requiring repeated interventions; thus offering symptomatic relief but no cure [10, 29–31]. Given its fibroinflammatory nature, an emerging concept of personalized immunotherapy approach has been explored for the treatment of FM. Westerly et al. reported that rituximab alleviated symptoms and reduced lesion size in FM patients whose biopsy specimens showed B lymphocyte infiltration, highlighting the potential benefits of a targeted anti-inflammatory strategy tailored to individual immunological profiles [32]. In line with previous studies demonstrating that samples from mediastinal cryobiopsy facilitate immunological testing in lung cancer, we showed that large cryosamples with preserved architecture enable a comprehensive and individualized assessment of FM-PH immunophenotyping [14, 15]. In addition to the known increase in B cell infiltrates, immunostaining of cryo-samples revealed an abundance of other immune subsets, including CD4^+^ and CD8^+^ T cells as well as macrophages, which comprised the major immune cell population within or surrounding the fibrotic lesions, indicating their potential roles in the inflammatory disorder of FM-PH. Therefore, samples from cryobiopsy might provide a foundation for in-depth exploration of disease pathogenesis and for the identification of potential therapeutic targets of FM-PH.

This study has limitations. First, the sample size of our study is relatively small, primarily due to the rarity of FM-PH. To the best of our knowledge, this is the first cohort study to assess the clinical usefulness of EBUS procedures in FM-PH. Second, the single-center retrospective design of this study might introduce selection bias, limiting the generalizability and applicability of our findings. Larger, multicenter prospective studies are warranted to validate the diagnostic performance and safety of EBUS-guided biopsy. Third, while immunohistochemical analyses provided valuable insights, their clinical significance in the pathogenesis and treatment of FM-PH requires further investigation.

## Conclusion

In conclusion, transbronchial mediastinal cryobiopsy might serve as a diagnostic choice for patients with FM-PH. Future research with larger cohorts is needed to further establish the safety and indications of this technique in FM-PH, as well as to clarify its potential for guiding personalized treatment.

## Supplementary Information

Additional file 1.

## Data Availability

All data generated or analyzed during this study are included in this article. Further enquiries can be directed to the corresponding authors.

## Abbreviations

FM: Fibrosing mediastinitis
FM-PH: Fibrosing mediastinitis-associated pulmonary hypertension
EBUS: Endobronchial ultrasound
EBUS-TBNA: Endobronchial ultrasound-guided transbronchial needle aspiration
