# Supplementary material for: Ultrasound-guided transbronchial biopsy in the diagnosis of fibrosing mediastinitis-associated pulmonary hypertension
Source: Orphanet J Rare Dis. 2025 Apr 15;20:180. doi: 10.1186/s13023-025-03695-3 (PMC12001500; doi:10.1186/s13023-025-03695-3)
Supplement: Supplementary file 1 — Additional file 1. [file 13023_2025_3695_MOESM1_ESM.docx]

**Supplement**

**Materials and methods**

**Human samples**

Studies using human samples were conducted in accordance with the principles outlined in the Declaration of Helsinki and were approved by the Institutional Review Boards of the Third Military Medical University in Chongqing, China (2019-062-01). All participants provided written informed consents.

**Needle aspiration**

The most suspicious target (mediastinal lesion with disease characteristics suggested by imaging data, such as significantly enlarged size, contrast enhancement on CT imaging, high FDG uptake on PET/CT imaging, among others) with relatively lower biopsy risk was selected for sampling. The site for needle aspiration was determined using EBUS guidance. Once the target lesion is visualized via ultrasound, a dedicated needle for transbronchial aspiration (either 21-gauge or 22-gauge, NA-201SX-4021 or NA-201SX-4022, Olympus, Tokyo, Japan) is inserted through the working channel of the EBUS bronchoscope. Under real-time ultrasound guidance, the needle is advanced through the tracheobronchial wall into the target lesion. Care is taken to avoid areas with high blood supply or extensive necrosis. After removing the central stylet, suction is applied using a syringe, while the needle is moved back and forth 25 times within the lesion. The collected specimen is expelled from the needle's lumen and transferred onto a glass slide with air using a syringe. The specimen on the slide is then smeared and fixed in 95% alcohol for cytological examination. The visible tissue fragment on the glass slide, along with any residual specimen in the needle's lumen, are collected and placed in formalin-filled containers for cell block analysis. Both cell block and glass slide-based cytology analyses are performed. Needle aspirations were repeated for 4 times.

**Immunohistochemistry**

For tissue Immunohistochemistry, sections were deparaffinized and rehydrated by serial immersion in xylene, alcohol (95%, 85% and 75%) and water. Afterwards, the tissues were incubated for 10 minutes with 3% hydrogen peroxide to block endogenous peroxidase activity, followed by blocking nonspecific signals by 5% BSA. The sections were incubated with primary antibodies against CD3, CD4, CD8, CD20, CD56, CD68, BCL6, FOXP3, and PD-1 overnight (**Table 1**). The horseradish peroxidase-conjugated goat-anti-mouse (or rabbit) IgG antibody was incubated with sections for 1 hour at room temperature and labeled by diaminobenzidine tetrahydrochloride. Cell nuclei were counterstained with hematoxylin for morphological observation.

**Statistical analysis**

Data were expressed as mean ± SD and analyzed using PASW Statistics 27 (SPSS Inc., Chicago, IL, USA). Statistical comparisons were conducted using Chi-squared test. A p-value of <0.05 was considered statistically significant.

**Table I. Antibodies for immunoblot analyses**

| Antibody | Manufacturer | Catalog | Source of species |
| --- | --- | --- | --- |
| CD3 | Servicebio | GB11014-50 | rabbit |
| CD4 | Proteintech | 19068-1-AP | rabbit |
| CD8 | Servicebio | GB12068-100 | mouse |
| CD20 | CST | 48750S | rabbit |
| CD56 | CST | 3576S | mouse |
| CD68 | Servicebio | GB113109 | rabbit |
| BCL6 | CST | 14895S | rabbit |
| FOXP3 | Servicebio | GB112325 | rabbit |
| PD-1 | CST | 86163S | rabbit |
